# Patients' Responses to COVID-19 Pandemic: The Relationship Between Potential Pandemic-Induced Disruptions, Ontological Security, and Adaptive Responses in Taizhou, China

**DOI:** 10.3389/fpubh.2022.865046

**Published:** 2022-05-19

**Authors:** Chengwen Luo, Xiaoyan Wu, Weizhen Wang, Mei-Xian Zhang, Fengmin Cheng, Haixiao Chen, Tao-Hsin Tung

**Affiliations:** ^1^Evidence-Based Medicine Center, Taizhou Hospital of Zhejiang Province, Wenzhou Medical University, Linhai, China; ^2^Taizhou Hospital of Zhejiang Province, Wenzhou Medical University, Linhai, China; ^3^Department of Nursing, Taizhou Hospital of Zhejiang Province, Wenzhou Medical University, Linhai, China; ^4^Department of Orthopedics, Taizhou Hospital of Zhejiang Province, Wenzhou Medical University, Linhai, China

**Keywords:** ontological security, pandemic-induced disruption, pandemic control, adaptive practice, COVID-19

## Abstract

**Background:**

The COVID-19 pandemic has changed the social environment of most individuals around the world and has profoundly impacted people's lives, ontological security, and behavior. Among them, the patients are one of the groups most influenced by the pandemic.

**Objective:**

The present research aimed to study the relationship of COVID-19 pandemic-induced disruption to patients' daily lives, ontological security, and patients' responses to prevent the spread of COVID-19, and explore the role of ontological security.

**Methods:**

This article was based on an online structured questionnaire study conducted among hospitalized patients in Taizhou Hospital of Zhejiang Province, Taizhou, China, from 8 July to 11 August 2021. We analyzed the data using the multivariate regression model and mediation analysis method.

**Results:**

The results showed that the higher the pandemic-induced disruption to inpatients' lives, the better behavior would be taken by hospitalized patients to prevent the spread of COVID-19, and the perceived scarcity of ontological security played a mediating role in this process. Higher pandemic-induced disruption to patients' lives increased the ontological insecurity which further, in turn, reduced patients' good practice toward measures to prevent the novel coronavirus.

**Conclusion:**

These findings provided direct evidence for the relationship between pandemic-induced disruption, scarcity of ontological security, and patients' prevention behavior. It suggested that there was a need to emphasize patients' ontological security. Overall, these findings suggested that it is important to emphasize the mental health among patients during the COVID-19 pandemic, and implement strategies to offer psychological support when needed.

## Background

The coronavirus disease 19 (COVID-19), since its first outbreak in late 2019, has been a major public health exigency of international concern. The pandemic outbreak has posed a negative influence on the economy and health, and dramatically changed people's social and living environment, in addition to a threat to most of the patients worldwide (1–3). For instance, people were required to stay at home and maintain social distance for an extended period; in addition, discrimination, and violence against Asian and minority groups posed by the COVID-19 epidemic also broke out in many countries (4–6). Meanwhile, the COVID-19 pneumonia pandemic has led to severe disruptions to people's lives and also has caused various psychological problems of panic and anxiety (7).

The COVID-19 pandemic disrupted people's daily lives, which cut people off from social interactions, including sharing family weekends and going out to a restaurant. At the same time, the world was experiencing health and social disruption and unrest that few of us have seen in our lifetimes. Previous studies showed that such threat-induced disruptions could seriously disrupt the sense of ontological security and elicit adaptive responses among consumers (8). Ontological security proposed by Giddens was commonly applied to assess individuals' perception of sustainable stability of the environment and the psychological state of their continuous self-identity, which came from the psychological senses of people during the course of their interaction with the environment (9–11). Yang et al. showed that the pandemic-induced scarcity perception of ontological security could promote migrant workers' risk-taking tendency (12).

During the peak period of the pandemic, the fulfillment of national policies to keep away from social gatherings and travel to any high-risk area resulted in hospitals reducing patient clinics, and many services were limited to urgent cases. Facing such a global COVID-19 epidemic, a variety of protective measures have been adopted to prevent the spread of the COVID-19 virus, including washing hands, using facial masks, and keeping social distance. Nevertheless, scare attention has been paid in the study of the mechanism of pandemic-induced disruption on patients' behavior to prevent the transmission of the COVID-19 pneumonia epidemic.

In this study, we aimed to explore the relationship between the COVID-19 pandemic caused disruption, ontological insecurity, and adaptive responses. We adopted the mediation analysis approach to investigate the above relation. The exposure we considered here was pandemic-induced disruptions to lives; the potential mediator was the ontological insecurity; and the outcome was the good practice taken by the inpatients to prevent the spread of the COVID-19 epidemic.

## Methods

### Study Design

An online questionnaire was designed to survey the hospitalized patients at a tertiary hospital in Taizhou, China, from 8 July to 11 August 2021. The anonymous cross-sectional survey was conducted *via* the WeChat-incorporated Wen-Juan-Xing Platform, which was the largest online survey platform in China. In this survey, our target population was hospitalized patients in Taizhou Hospital of Zhejiang Province during the study period. The participants received the questionnaire *via* e-mail or WeChat and the interviewees answered the self-administered survey by scanning the Quick Response code or visiting the Uniform Resource Location on their mobile phones. This research was reviewed and approved by the Ethics Committee of Taizhou Hospital of Zhejiang Province (Approval Number: K20210521) in China. All procedures were performed following the guidelines of our institutional ethics committee and adhered to the tenets of the Declaration of Helsinki. All participants provided informed consent to participate. All interviewees' information was anonymous.

The questionnaire incorporated the following relevant components: 1) the demographic data of the interviewees, such as gender, age, address, education, and occupation; 2) the COVID-19 pneumonia epidemic induced disruptions on daily lives including a total of eleven items, e.g., health, economic, social, information, environment, work, spending/saving, social lives and identity, rituals and practices, institutions, and beliefs; 3) the test of ontological insecurity *via* nine items (13, 14), such as “I have no identity of my own; my identity is shaped by how others see me” and “Sometimes I cannot recognize myself when I look in the mirror”; and 4) the good practice taken by the inpatients to prevent the spread of the novel coronavirus, including washing hands, wearing facial masks, and application of social distancing. More details about the questionnaire could refer to the Supplementary Material.

### Study Participants

A total of 1,223 hospitalized patients volunteered to participate and completed the questionnaire from 8 July to 11 August 2021. Patients younger than 18 years old were excluded. In addition, samples that took <2 min to answer the questionnaire were also excluded. Moreover, repeat responses were subject to the first submission. Ultimately, of 1,223 participants, 1,185 responses were valid, with a valid response rate of 96.9%. The average age of hospitalized patients was 51.7 years (SD = 16.6), and there were 542 males (45.7%) and 643 females (54.3%).

### Measures

Coded data were imputed into MS Excel and scored based on the Likert scale method. Scored responses were summed and used *via* calculating the total scores of pandemic-induced disruptions, ontological insecurity, and good practice of inpatients during the COVID-19 epidemic for each participant.

Take ontological insecurity as an example, we adopted the Likert scale method to give 0~4 points for each response given by each inpatient from very disagree, disagree, neutral, agree, and very agree, respectively. In total, the scores of these 9 items of ontological insecurity were added up in a range of 0–36 points. The larger the value was, the less the security of ontology was. The scores of pandemic-induced disruptions and behavior of the inpatients were calculated similarly. For pandemic-induced disruptions, we also gave 0–4 points for each response given by each hospitalized patient from very disagree, disagree, neutral, agree, and very agree, respectively. In total, the scores of the 11 items of pandemic-induced disruptions were ranged 0–44 points, and the median value was 28. We measured the disruptions as high and low levels based on the median value as the cutoff. Besides, the scores of the 26 items of the behavior were ranged 0–78 points. Here, we adopted the Likert scale method to give 0–3 points for each response given by each inpatient from always, often, occasionally, and never, respectively.

### Mediation Analysis

Mediation models have been broadly utilized to investigate the potential mechanism of an independent variable on a response variable, and whether there was a variable that mediated the above relationship (15, 16). Based on mediation analysis, researchers could easily partition the total effect of an independent variable on an outcome variable into direct and indirect effects *via* an intermediate variable (17, 18). This could have important policy consequences since mediation analysis played an important role in understanding the potential mechanism whereby the change in one variable caused the change in another. In this study, the exposure (*X*) we considered here was pandemic-induced disruptions to lives; the potential mediator (*M*) was the ontological insecurity; and the outcome (*Y*) was the good practice taken by the inpatients to prevent the spread of the COVID-19 epidemic.

We focused on when we had a continuous outcome (*Y*) and a continuous mediator (*M*). We considered the following three regression models for mediation analysis:


(1) $$\begin{array}{lll} Y & = & c_{1}+\gamma X+\delta^{T}Z+\varepsilon_{1}, \end{array}$$

(2) $$\begin{array}{lll} M & = & c_{2}+\alpha X+\theta^{T}Z+\varepsilon_{2}, \end{array}$$

(3) $$\begin{array}{lll} Y & = & c_{3}+\gamma *X+\beta M+\vartheta^{T}Z+\varepsilon_{3}, \end{array}$$

where Equation (1) described the relation of the exposure and the outcome (*X & Y*); Equation (2) characterized the relation of the exposure and the mediator (*X & M*); Equation (3) summarized the relationship between the exposure, the mediator, and the outcome (*X, M & Y*); *Z* was the covariates, such as age and gender; γ was the total effect of the exposure on the outcome; α was the effect of the exposure on the mediator; γ^*^ was the direct effect of the exposure on the outcome; β was the effect of the mediator on the outcome; *c*_1_, *c*_2_, and *c*_3_ were the intercept terms; ε_1_, ε_2_, and ε_3_ were the residual terms.

Mediation was usually tested by a regression-based modeling method (19–21). The first step was to identify whether there was a significant relationship between the exposure *X* and the outcome *Y*. The second step was to determine whether the relation of the exposure *X* and the mediator *M* was significant. The final step was to regress the outcome *Y* on both *X* and *M*. In addition, to test the mediation effect, we applied the joint test method (22). This approach used the path-specific *P*-values and did not provide an estimate as follows


$$\begin{array}{l} P=\max\left(P_{\alpha },P_{\beta }\right). \end{array}$$

Thus, we could conclude that the variable *M* was the intermediator between the exposure and the outcome if *P* < 0.05.

### Statistical Analysis

**Figure 1:**
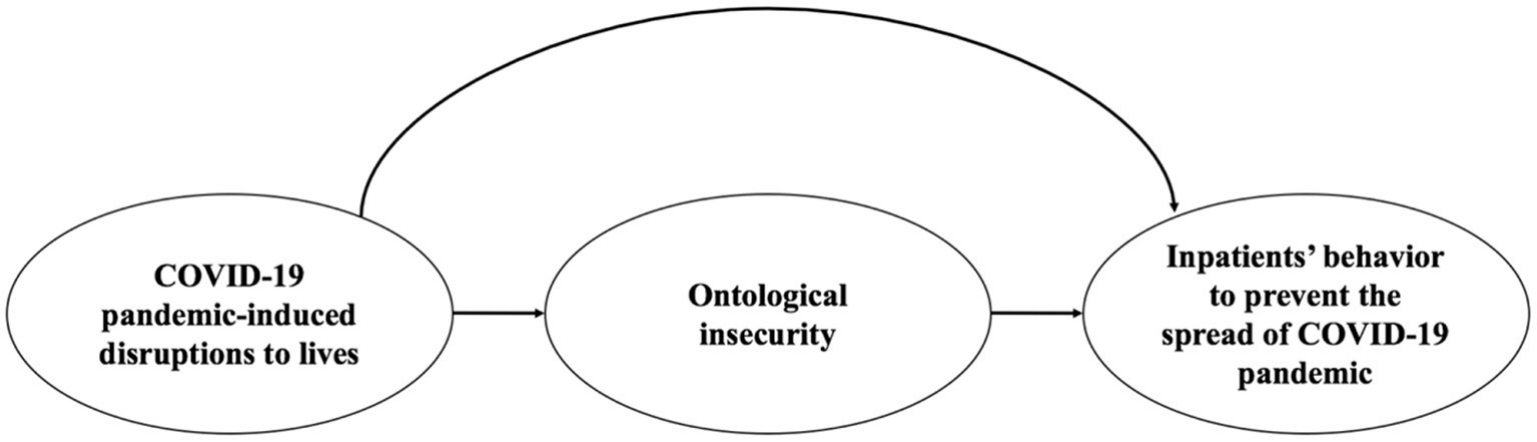
A framework of the relationship between the COVID-19 pandemic-induced disruptions to inpatients' lives, ontological insecurity, and patients' behavior to prevent the spread of the COVID-19 pandemic.

In this study, our main purpose was to explore the relationship between the disruptions created by the COVID-19 pandemic to hospitalized patients' lives, ontological security, and inpatients' adaptive behavior. The framework of the above relationship was characterized in Figure 1. The exposure we considered here was the pandemic-induced disruptions; the potential mediator was the ontological security; and the outcome was hospitalized patients' behavior. We conducted mediation analysis based on the above mediation models and adjusted for covariates including age, gender, address, education, and occupation.

Categorical variables of the basic demographic characteristics were presented as counts and percentages. We applied the chi-square test to initially identify the possible factors of the outcome. Finally, we adopted the three regression models [i.e., Equations (1)–(3)] to conduct the mediation analysis. Variables considered statistically significant should have a *P*-value < 0.05. All statistical analyses were implemented *via* R software, version 4.1.0 (R Project for Statistical Computing).

## Results

### Basic Characteristics of the Participants

In this study, a total of 1,223 hospitalized patients completed the survey, with 1,185 responses being valid. The basic demographic characteristics of the subjects were shown in Table 1. The average age of 1,185 hospitalized patients was 51.7 years (SD = 16.6), with the majority between 30 and 60 years old (52.6%). Among all the respondents, there were 542 men (45.7%) and 643 women (54.3%). The proportion of respondents from rural was 53.1%, which was higher than those from the city (21.3%). Besides, most of the participants had a primary school education (41.1%), and most of them were farmers (39.2%).

**Table 1:** Baseline characteristics of the patients (*N* = 1,185).

**Characteristics**	**Category**	**Sample**	
		**Number**	**Percentage (%)**
Age (years)	<30	164	13.8
	30–60	623	52.6
	>60	398	33.6
Gender	Male	542	45.7
	Female	643	54.3
Address	Rural	629	53.1
	Villages & Towns	303	25.6
	City	253	21.3
Education	Primary and below	487	41.1
	Junior secondary	343	28.9
	Senior secondary	171	14.4
	College	176	14.9
	Graduate	8	0.7
Occupation	Farmer	465	39.2
	Blue-collar	99	8.4
	White-collar	184	15.5
	Others	437	36.9

*“Others” for occupation includes freelancers, the self-employed, and the unemployed*.

Table 2 demonstrated that the practice score of daily epidemic prevention post by the subgroup of participants. The mean (SD) behavior score of the sample was 60.9 (14.9). People under 30 years had the highest practice score [mean (SD) = 68.8 (11.3)], while individuals between 30 and 60 years of age have a lower practice score [mean (SD) =61.4 (14.7)], and those above 60 have the lowest practice score [mean (SD) = 57.0 (15.2), *P*-value < 0.001]. Female respondents showed significantly higher good practice in preventing the spread of COVID-19 than male [mean (SD) = 62.2 (14.7) vs. 59.4 (15.0), *P*-value = 1.1E−03]. Compared to inpatients from the city, those from rural have lower practice scores [mean (SD) = 58.5 (15.4)]. Meanwhile, individuals with a college and above education have the highest practice score [mean (SD) = 67.6 (11.6)], while those with a primary school education have the lowest practice score [mean (SD) = 55.9 (15.6)]. It is noteworthy that farmer participants experienced the lowest level of practice score [mean (SD) = 56.7 (15.3), *P*-value < 0.001] among all occupations. Overall, the results of univariate analysis recommended that pandemic-induced disruption, age, gender, address, education, and occupation were factors influencing inpatients' behavior.

**Table 2:** Univariate analysis of factors associated with patients' behavior.

**Variables**	**Category**		**t/F**	***P*-value**
Total		60.9 (± 14.9)		
Disruption			25.2	5.9E−07
	Low^*^	58.6 (± 15.7)		
	High	62.9 (± 13.9)		
Age (years)			39.0	<2.0E−16
	<30	68.8 (± 11.3)		
	30–60	61.4 (± 14.7)		
	>60	57.0 (± 15.2)		
Gender			10.8	1.1E−03
	Male	59.4 (± 15.0)		
	Female	62.2 (± 14.7)		
Address			36.8	1.8E−09
	Rural	58.5 (± 15.4)		
	Villages & Towns	62.7 (± 14.4)		
	City	64.7 (± 13.3)		
Education			45.9	<2.0E−16
	Primary and below	55.9 (± 15.6)		
	Junior Secondary	61.4 (± 14.4)		
	Senior secondary	67.2 (± 11.5)		
	College and above	67.6 (± 11.6)		
Occupation			29.6	<2.0E−16
	Farmer	56.7 (± 15.3)		
	Blue-collar	58.7 (± 14.9)		
	White-collar	67.3 (± 12.3)		
	Others	63.2 (± 14.2)		

* *High and low groups were divided by the median value of pandemic-induced disruption*.

### Testing of the Mediation Model

**Figure 2:**
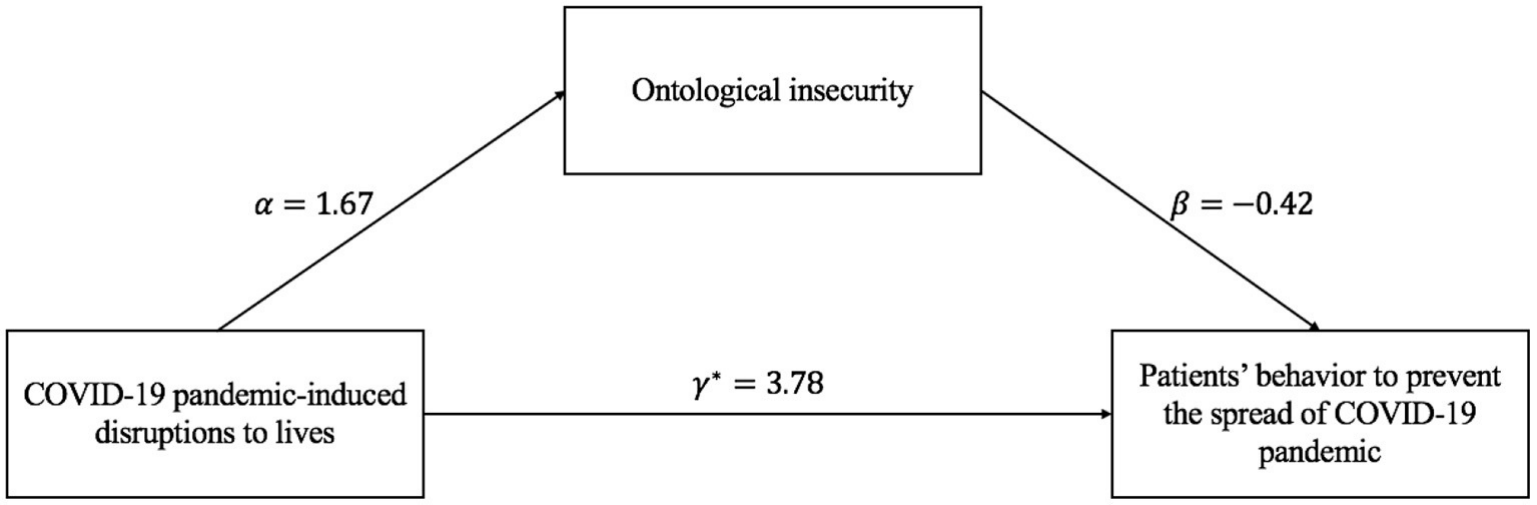
Pathway between COVID-19 pandemic-induced disruptions, ontological insecurity, and patients' behavior.

The results of the mediation model exploring the relationship of the COVID-19 pandemic-induced disruption to hospitalized patients' lives, ontological insecurity, and patients' adaptive behavior were presented in Table 3 and Figure 2. These regression results were adjusted for age, gender, address, education, and occupation.

**Table 3:** Model test of mediating effect of ontological insecurity.

**Variable**	**Model 1**		**Model 2**		**Model 3**	
	***B***	***SE***	***B***	***SE***	***B***	***SE***
*Independent variable*						
Disruption (High vs. Low)	3.07^***^	0.82	1.67^***^	0.46	3.78^***^	0.80
*Mediator*						
Ontological insecurity					−0.42^***^	0.05
*Controlled variable*						
Age (<30)						
30–60	−2.99^*^	1.40	−0.96	0.78	−3.40^*^	1.36
>60	−3.49^*^	1.68	−1.15	0.94	−3.97^*^	1.64
Gender (Male)						
Female	1.55	0.84	0.05	0.47	1.57	0.82
Address (Rural)						
Village & Town	3.11^**^	0.98	−1.18^*^	0.55	2.61^**^	0.96
City	2.64^*^	1.12	−0.98	0.63	2.22^*^	1.09
Education (Primary)						
Junior secondary	4.14^***^	1.11	0.52	0.62	4.36^***^	1.08
Senior secondary	7.81^***^	1.50	−0.87	0.83	7.44^***^	1.46
College and above	6.49^***^	1.74	−1.81	0.97	5.73^***^	1.70
Occupation (Farmer)						
Blue-collar	−1.24	1.62	0.34	0.91	−1.10	1.58
White-collar	3.16^*^	1.59	0.95	0.89	3.57^*^	1.54
Others	1.47	1.09	0.79	0.61	1.81	1.06

* *P-value < 0.05*;

** *P-value < 0.01*;

*** *P-value < 0.001. The outcome of Model 1 and 3 was patients' behavior to prevent the transmission of the COVID-19 pneumonia pandemic; the outcome of Model 2 was ontological insecurity*.

Firstly, the effect of COVID-19 pandemic-induced disruption to hospitalized patients' lives on patients' behavior was significant. Compared with the low disaster-induced disruption to inpatients' lives, better practice would be taken by those with high disruption created by the COVID-19 pandemic to prevent the novel coronavirus. Besides, compared with participants with lower impact from the pandemic-induced disruption, those higher were more likely to have higher ontological insecurity. Further, the effect of ontological insecurity on patients' behavior was −0.42, which denoted that the less sense of ontological security, the worse the prevention and control behavior of patients. Finally, the results of the joint test illustrated that the mediation effect of ontological insecurity on the relationship between the COVID-19 pandemic-induced disruptions to hospitalized patients' life and patients' adaptive behavior was significant [*P* = max(*P*_β_α__, *P*_β_) < 0.05]. Furthermore, as presented in Figure 2, the direct effect of COVID-19 pandemic-induced disruptions on the inpatients' adaptive behavior was 3.78, while the indirect effect which mediated by ontological insecurity was αβ = −0.71. We could get the conclusion that ontological insecurity could significantly mediate the effect of the COVID-19 pandemic-induced disruption to the lives of inpatients on inpatients' adaptive behavior.

## Discussion

The aim of this research was to explore the influence of the COVID-19 pandemic-caused disruptions to lives on hospitalized patients' behavior and the potential mechanism. This study focused on hospitalized patients, since they were vulnerable to the pandemic. Most of these inpatients were farmers and came from rural with low education levels. During the pandemic, not only did life threaten, but it was also more susceptible to disruption. Therefore, whether and how disaster-induced disruptions impacted their practice taken to prevent the transmission of the COVID-19 might influence the stability of hospitals and even the society, which was also a key topic that the governments and hospitals wanted to focus on. To our best knowledge, this paper was one of the few researches on the influence of disruptions to lives caused by the COVID-19 epidemic on the behavior of hospitalized patients.

In this study, through the online survey, it was shown that the epidemic-induced disruptions to lives had a significant impact on the good practice of hospitalized patients to prevent the COVID-19 pandemic. It could be seen that the more serious the inconvenience and disturbance caused by the COVID-19 epidemic to the inpatients' lives, the better the prevention and control behavior of patients will be standardized. However, the more serious the inconvenience and disturbance brought by the epidemic to the inpatients, it would increase the ontological insecurity of inpatients, thus, in turn, reducing the good prevention and control behavior of patients. Previous studies have reported that the lack of ontological security could promote people's risk-taking tendency among migrant workers (12). In addition, the research in Australia illustrated that the concerns about the uncertainties of the future created by the COVID-19 crisis might trigger ontological insecurity (23).

Overall, this research explored the relationship between threat-induced disruptions, ontological security, and adaptive behavior of hospitalized patients during pandemic situations. Previous researches pointed out that people in resource-scarce environments would present a higher degree of psychological insecurity and chose a fast survival strategy which was generally more inclined to violate social norms and choose high-risk programs (24–26). The research results of this study also supported the above conclusions, that during the COVID-19 epidemic, the pandemic-induced disruptions would regulate people's behavior, but also increased the scarcity perception of ontological security which in turn led to choosing more risk-taking tendencies. Meanwhile, the COVID-19 epidemic has undoubtedly threatened the health of individuals and caused great disruption to the lives of individuals around the world, which promoted individuals to pay more attention to take good behavior in the health field during the epidemic.

The prolonged lockdown related to COVID-19 pandemic determined disruption of lifestyle and social isolation. The pandemic has adversely affected mental health, especially in individuals with chronic disease such as Parkinson's disease, relapsing-remitting multiple sclerosis patients, and patients with rheumatoid arthritis (27–29). This study was focused on the inpatients. However, due to nationwide regulations regarding commuting and the uncontrollable risk of infection, patients with chronic disease, such as Parkinson's which need regular outpatient visits for evaluations and prescriptions, had difficult in seeking medical treatment. The lack of medical consultation affected patients' mental health, such as higher depression scores than the healthy groups (27). It is important to emphasize the mental health among patients during the COVID-19 pandemic, and implement strategies to off psychological support when needed.

Although this research has certain practical value, however constrained to time and resource factors, there are several limitations. Firstly, this research mainly considered hospitalized patients, nevertheless, the relationship between the disruption, ontological security, and adaptive response created by the COVID-19 pandemic might also be applicable to the general public. Further researches should consider this selection bias, since there might be differences between hospitalized patients and the general public. Hence, future research should also consider the general public to explore to further investigate the mechanism of the pandemic-induced disruption on people's practice to prevent the spread of the epidemic. Secondly, though we have conducted the logic checks and called back for corrections of non-logic responses, the self-administered online survey could potentially lead to over-reporting or lower-reporting some indicators. Thirdly, due to the epidemic, we evaluated only one teaching hospital, and the number of survey samples in this research was not large enough. In addition, patients from different wards, such as hematology, cardiothoracic surgery, gastroenterology, and gastroenterology, could also affect their perception of COVID-19, which was also of future consideration. Therefore, in order to further explore the relationship between threat-induced disruptions, ontological insecurity, and adaptive responses, the generalization and external validity should be further studied. Fourthly, long-term exposure to the threat-induced disruptions, ontological insecurity, and adaptive responses might not be detected, since we conducted a single-time measurement. Besides, we performed the survey through an online questionnaire from July 8 to August 11, 2021. During this period, the COVID-19 epidemic was well contained in China. The degree of disruptions caused by the pandemic and ontological insecurity might be varied over time, which could also affect the results. Thus, in the future study, researchers should have another time point and hospitals to verify these findings. On the whole, it is of great importance for further studies to conduct a larger sample size or a long-term research over a wide range of regions, as the relationship might be influenced by the prevalence of the pandemic.

## Conclusion

In summary, our study showed that the greater disruptions to lives brought about by the COVID-19 crisis could promote the good practice taken by hospitalized patients to prevent the spread of the novel coronavirus. In addition, the pandemic-induced disruptions could also increase the ontological insecurity of hospitalized patients, which in turn promoted patients' risk-taking tendency. Overall, these findings suggested that it is important to emphasize the mental health among patients during the COVID-19 pandemic, and implement strategies to offer psychological support when needed.

## Data Availability Statement

The raw data supporting the conclusions of this article will be made available by the authors, without undue reservation.

## Ethics Statement

This study was reviewed and approved by the Ethics Committee of Taizhou Hospital of Zhejiang Province (Approval number: K20210521) in China. The patients/participants provided their written informed consent to participate in this study.

## Author Contributions

CL, HC, and T-HT conceived the idea, implemented the method, and drafted the manuscript. CL was responsible for the coding of the analyses. XW, WW, M-XZ, and FC collected the data. All authors edited and approved the final manuscripts.

## Funding

This paper was funded by National Natural Science Foundation of China (Funding ID: 72074189).

## Conflict of Interest

The authors declare that the research was conducted in the absence of any commercial or financial relationships that could be construed as a potential conflict of interest.

## Publisher's Note

All claims expressed in this article are solely those of the authors and do not necessarily represent those of their affiliated organizations, or those of the publisher, the editors and the reviewers. Any product that may be evaluated in this article, or claim that may be made by its manufacturer, is not guaranteed or endorsed by the publisher.
