# Supplementary material for: Patients' Responses to COVID-19 Pandemic: The Relationship Between Potential Pandemic-Induced Disruptions, Ontological Security, and Adaptive Responses in Taizhou, China
Source: Front Public Health. 2022 May 19;10:865046. doi: 10.3389/fpubh.2022.865046 (PMC9160831; doi:10.3389/fpubh.2022.865046)
Supplement: Supplementary file 1 [file Data_Sheet_1.pdf]

Supplementary Material

This questionnaire is conducted to study the behavior in epidemic prevention and control under the COVID-19 pandemic. The data collected by the questionnaire were only used for statistical analysis. This study was approved by the ethics committee. You have volunteered to participate in this survey.

- ## Basic Information

1. **Gender:** □ Male □ Female
2. **Date of Birth:** □□□□Year□□Month□□Day
3. **Educational Level:**

   □ Primary and Below □ Junior Secondary

   □ Senior Secondary □ College

   □ Graduate
4. **Occupation:**

   □ Staff □ Worker

   □ Farmer □ Others
5. **Address:**

   □ City □ Villages & Towns

   □ Rural

- ## The COVID-19 Epidemic Induced Disruptions on Daily Lives

6. The COVID-19 pandemic has affected you in the following ways:

	Strongly Disagree	Disagree	Neutral	Agree	Strongly Agree
1) Health					
2) Economic					
3) Social					
4) Information					
5) Environment					
6) Work or Study					
7) Spending or Saving					
8) Social Lives					
9) Rituals and Practices					
10) Institutions					
11) Beliefs					

- ## Ontological Insecurity

7. The test of ontological insecurity:

	Strongly Disagree	Disagree	Neutral	Agree	Strongly Agree
1) I have no identity of my own; my identity is shaped by how others see me					
2) Sometimes when I am completely alone, I wonder if I exist at all					
3) Sometimes I can't recognize myself when I look in the mirror					
4) I often feel that others are more real and alive than I am					
5) Sometimes I become fearful of events that others might not even notice					
6) Sometimes I experience myself as outside my body					
7) Others seem to relate in a more decisive, emphatic, and convincing manner than I ever could					
8) In the same moment, I can feel trapped between wanting to be with others and wanting to be completely alone					
9) My identity depends upon me being important to at least one other person					

- **Behavior**

8. How do you wash your hands?

	Always	Often	Occasionally	Never
1) Before processing food and beverage				
2) Care for the elderly and infants before				
3) Before you eat				
4) After touching public facilities (e.g., doorknobs, self-service machines, public bicycles)				
5) After touching the elevator button				
6) After using the toilet				
7) After hands covering for cough and sneeze				
8) There are significant contaminants in the hands				
9) After touching the money				
10) After contact with dirt (e.g., garbage cans)				
11) Wash your hands with running water				
12) Wash your hands with soap or hand sanitizer				
13) Wash your hands via six-step hand washing				

9. How do you wear facial masks?

	Always	Often	Occasionally	Never
1) When someone coughs or sneezes nearby				
2) When a stranger approaches				
3) When using public transport				
4) Enter crowded places (e.g., markets, stations, etc.)				
5) When entering indoor confined space (e.g., shopping mall, cinema, elevator, etc.)				

6) When someone reminds you to wear a mask				
7) Clean your hands, place the mask over your face, mouth and nose, and place the ropes over your ears				
8) When wearing a mask, press the metal strip on both sides of the bridge of the nose above the mouth and nose with the middle fingers of both hands to make it fit tightly				
9) Pull the folds of the mask up and down with both hands at the same time, ensuring full coverage of mouth, nose and chin				

10. How do you maintain social distancing?

	Always	Often	Occasionally	Never
1) Keep social distancing				
2) Avoid crowds				
3) Attend gatherings and dinners sparingly				
4) The queue stood one meter away				
